# *In vivo* study of anticancer activity of ginsenoside Rh2-containing arginine-reduced graphene in a mouse model of breast cancer

**DOI:** 10.22038/IJBMS.2022.66065.14524

**Published:** 2022-12

**Authors:** Shervin Dokht Farhangfar, Farzaneh Fesahat, Hadi Zare-Zardini, Mahdi Dehghan-Manshadi, Fateme Zare, Seyed Mohsen Miresmaeili, Maryam Vajihinejad, Hossein Soltaninejad

**Affiliations:** 1 Department of Biology, Science and Arts University, Yazd, Iran; 2 Reproductive Immunology Research Center, Shahid Sadoughi University of Medical Sciences, Yazd, Iran; 3 Hematology and Oncology Research Center, Shahid Sadoughi University of Medical Sciences, Yazd, Iran; 4 Department of Biomedical Engineering, Meybod University, Meybod, Iran; 5 Department of Pathology, Shahid Sadoughi University of Medical Sciences, Yazd, Iran; 6 Faculty of Interdisciplinary Science and Technology, Tarbiat Modares University, Tehran, Iran; # These authors contributed eqully to this work

**Keywords:** Arginine, Breast Cancer, Genes, Ginsenoside Rh2, Graphene, Tumor

## Abstract

**Objective(s):**

This study aims to evaluate the *in vivo* anticancer activity of arginine-reduced graphene (Gr-Arg) and ginsenoside Rh2-containing arginine-reduced graphene (Gr-Arg-Rh2).

**Materials and Methods:**

Thirty-two mice with breast cancer were divided into four groups and treated every three days for 32 days: Group 1, PBS, Group 2, Rh2, Group 3, Gr-Arg, and Group 4, Gr-Arg-Rh2. The tumor size and weight, gene expression (IL10, INF-γ, TGFβ, and FOXP3), and pathological properties of the tumor and normal tissues were assessed.

**Results:**

Results showed a significant decrease in *TGFβ* expression for all drug treatment groups compared with the controls (*P*=0.04). There was no significant difference among the groups regarding *IL10 *and *FOXP3* gene expression profiles (*P*>0.05). Gr-Arg-Rh2 significantly inhibited tumor growth (size and weight) compared with Rh2 and control groups. The highest survival rate and the highest percentage of tumor necrosis (87.5%) belonged to the Gr-Arg-Rh2 group. Lungs showed metastasis in the control group. No metastasis was observed in the Gr-Arg-Rh2 group. Gr-Arg-Rh2 showed partial degeneration of hepatocytes and acute cell infiltration in the portal spaces and around the central vein. The Gr-Arg group experienced a moderate infiltration of acute cells into the port spaces and around the central vein. The Rh2 group also showed a mild infiltration of acute and chronic cells in portal spaces.

**Conclusion:**

Based on the results, Gr-Arg-Rh2 can reduce tumor size, weight, and growth, TGF-β gene expression, and increase tumor necrosis and survival time in mice with cancer.

## Introduction

Breast cancer is the most common type of cancer with a high prevalence in women. This type of cancer is the second most common among women (1). There are several treatment methods for breast cancer, such as surgery, chemotherapy, hormonal therapy, biological therapy, and radiation therapy, among which chemotherapy is the most common. The type of treatment depends on the kind of breast cancer and how far it spreads from the breasts. Patients with breast cancer often undergo more than one kind of treatment (2-4). Aside from its positive effects, chemotherapy is associated with different mild and serious side effects, namely normal tissue damage (i.e., lung, heart, gonads, kidney, nerve system, etc.), nausea, vomiting, diarrhea, hair loss, loss of appetite, fatigue, fever, mouth sores, pain, constipation, easy bruising, bleeding, etc. (5, 6). Natural compounds with herbal origins hold key roles in pharmaceutical sciences. Various herb-derived agents have been developed for cancer treatment (7, 8). Ginsenoside Rh2 (Rh2), known as a natural compound, is isolated from *Panax ginseng*. Rh2 has demonstrated a potent anticancer activity and inhibition of proliferation, invasion, and metastasis. This agent can also reduce chemotherapy-induced side effects (9-11). Nevertheless, the low oral bioavailability and fast plasma elimination have limited the application of this agent as an anticancer drug (12). Nano-based drug delivery systems can improve efficacy and reduce the side effects of anticancer drugs (13, 14). Carbon nanostructures, especially graphene and its derivatives, have proper usage properties as a drug delivery system. Graphene with a high surface area and unique physical and chemical properties can be used as an excellent drug delivery system. Reduced graphene (rG), as an important derivative of graphene, is a form of processed graphene oxide (GO) with reduced oxygen content. This type of graphene is known as crumpled graphene (15-18). The specific surface area, conductivity, and stability against aggregation increase during rG synthesis. These properties lead to enhancement of drug loading capacity (19). The addition of active functional groups such as basic amino acids (Lys and Arg) can improve these interesting properties to a greater extent (17). Zare-Zardini *et al*. showed that functionalization of Arg-reduced graphene with Rh2 (Gr-Arg-Rh2) could help overcome the problems associated with drug instability. These researchers showed that Gr-Arg and Gr-Arg-Rh2 have more inhibitory effects on the growth of cancer cells in comparison with normal cells. On the other hand, the presence of Arg led to a significant reduction of side effects of functionalized graphene in comparison with non-functionalized graphene, where increased therapeutic index of Rh2 (stability enhancement, synergism anticancer activity, and slow-release) and reduced side effects on normal cells are advantages of Gr-Arg-Rh2 (20). Given that these researchers indicated that Gr-Arg-Rh2 could exert considerable anticancer activity on MDA-MB breast cancer cells *in vitro*, Gr-Arg-Rh2 was tested as an anticancer agent in an animal model with breast cancer in this study. This study was designed to assess the effect of Gr-Arg and Gr-Arg-Rh2 in treating mice with breast cancer. The effect of Gr-Arg and Gr-Arg-Rh2 on gene expression and the histology of the tumor and vital tissues were also investigated after the drug treatment.

## Materials and Methods

***Chemicals and instruments***

Rh2, arginine, and graphite were purchased from Sigma Company. Chemical agents were purchased from Merck Inc. Fourier-transform infrared (FTIR) and Raman spectroscopy were used to confirm the final characteristics of synthesized drugs. High-resolution transmission electron microscopy (HRTEM) was applied for morphological examination. Industrial microwave systems were utilized for nano-functionalization. Female BALB/c mice (aged 5–6 weeks) were obtained from Pasteur Institute (Iran). In addition, 4T1 cells (mice mammary gland tumor cells) were purchased from Pasteur Institute (Tehran, Iran). These cells were used for tumor induction in the animal model. 

***Ethical consideration***

All experimental protocols were approved by the Ethical Committee of Shahid Sadoughi University of Medical Sciences, Yazd, Iran (Project code: IR.SSU.fm.REC.1397.42). All ethical indicators were observed in working with animals. The conditions of keeping the mice were suitable, and they were provided with the right food and water.

***Drug system synthesis and characterization***

**Figure 1:**
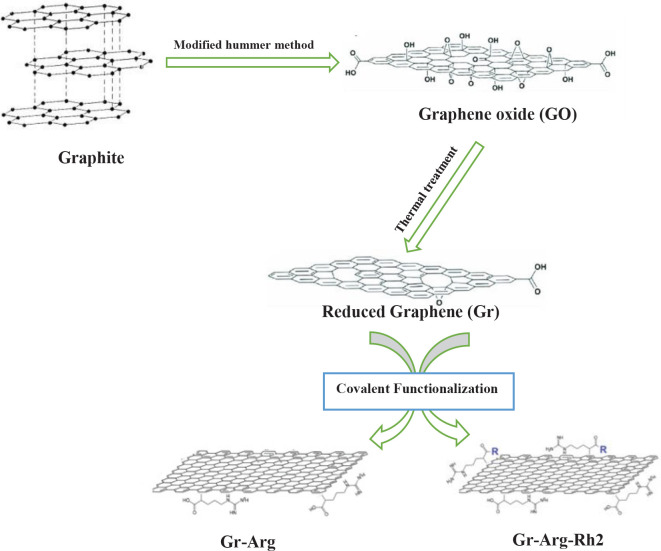
Schematic diagram of the synthesis procedure of graphene and functionalized graphene with Arginine and Rh2

Similar to our previous study (20), Rh2-containing arginine-treated graphene (Gr-Arg-Rh2) and arginine-treated graphene (Gr-Arg) were synthesized by the microwave technique (Figure 1). 

UV spectroscopic measurement was applied to analyze drug loading efficiency (Encapsulation Efficiency, EE). The nanostructure-containing solution was centrifuged at 12000 g for 10 min. Then, the supernatant was collected and analyzed by UV spectrophotometer at 232 nm (λmax) for estimation amounts of unloaded Ginsenoside Rh2. For %EE calculation, the below equation was used:

%EE= Total amount of Rh2 mg-unloaded Rh2 (mg)Total amount of Rh2 mg×100

We also evaluated this for *in vitro* drug release. At this step, nanostructure-containing solutions were prepared in a dialysis bag immersed in phosphate-buffered saline (PBS, pH 7.5). This apparatus was continuously stirred for 72 hr, and the drug release from the designed nanostructures was evaluated. 

One milliliter of PBS surrounding the dialysis bag was collected and analyzed by UV/visible spectroscopy at 232 nm to evaluate absorbance intensity at pre-specified interval times (0.5, 1, 2, 4, 6, 8, 12, 24, and 48 hr).

***Tumor induction in an animal model***

Thirty-two mice were included in this study. Inclusion criteria for animal selection were: female Balb/c mice, mean weight 22–30 gr, and age of 5–6 weeks. Considering confidence interval=0.95, power=0.8, SD=0.25, mean weight of tumor for the control group=2.9, and mean weight of tumor for Nano drug=1.2 (21), the minimum sample size was calculated as 8 using PASS15. 

All mice were kept in cages under standard conditions (12-hour light/dark cycle at 22–24 °C). Based on a similar study, these mice were injected with 5× 105 4T1 cells/100 μl/mice in their left flanks for tumor induction (22). Ten days following the tumor cell injection, the initial tumor was palpable. Non-tumorized mice were excluded from the study after tumor induction and replaced with suitable mice with the appropriate inclusions mentioned above.

***Drug therapy***

This study used four types of therapies: PBS, Rh2, Gr-Arg, and Gr-Arg-Rh2. Ten days after tumor cell injection and appearance of a palpable mass, the tumorized mice were divided into four study groups as follows:

Group 1) PBS as placebo controls (N=8), group 2) Rh2 (N=8), group 3) Gr-Arg (N=8), and group 4) Gr-Arg-Rh2 (N=8).

Before the first injection, all mice were weighed, and the tumor size was recorded in millimeters. Then, all mice were given intravenous injections according to the protocol suggested in similar studies as follows: 

Group 1, PBS: 60 µl (6 mg/kg) 

Group 2, Rh2: 60 µl (6 mg/kg) 

Group 3, Gr-Arg: 70 µl (3 mg/kg) 

Group 4, Gr-Arg-Rh2: 70 µl (3 mg/kg) 

The mice were given intravenous injections of the drug or a placebo every three days and ten times during 32 days. The mice were followed up until day 50 after 4T1 cell injection. 

Tumor size was measured every three days. The mice were euthanized with sodium pentobarbital if their weight loss was <15% of their initial weight or the tumor size was ≥1000 mm3. On day 50, all the surviving mice were discarded. The average tumor weight was calculated in each group. 

Four critical variables related to the drug effect in mice were calculated and compared:

1. The time to reach the end-point (TTE) of the tumorized mice in each group 

2. The percent of tumor growth delay (%TGD) = [(T − C)/C] × 100

3. The median survival time (MST) 

4. The frequency of increased life span (%ILS) = MST of treated group/MST of buffer group ×100 – 100 (23). 

***Morphological study***

Light microscopy was used to examine mice’s tumor, lung, and liver tissues. After animal euthanasia by sodium pentobarbital and removing the tissues, each tissue was cut into equal blocks and embedded in paraffin. For further analysis, a serial section of 5 mm thickness was cut from each block and stained with hematoxylin-eosin (H&E).

***Gene expression assessment***

According to the kit instructions, the total RNA was extracted from mice’s spleen tissue (Pars Tous biotechnology, cat no: A101231). The quantity and purity of each RNA were measured using a UV-spectrophotometer (PhotoBiometer, Eppendorf, Germany). The total RNA was transcribed reversely into the single-stranded complementary DNA (cDNA) according to the manufacturer’s kit protocol (Parstous biotechnology, cat no: A101161) and in the presence of oligo (dT) and random primer sets. The expression profiles of forkhead box P3 (*Foxp3*), transforming growth factor-β (*TGF-β*), interleukin 10 (IL10), and interferon-gamma (*IFNγ*) were determined using SYBR Green PCR Master Mix (Applied Biosystems, USA). The glyceraldehyde-3-phosphate dehydrogenase (GAPDH) gene was used as a housekeeping gene (Table 1). The real-time quantitative polymerase chain reaction (RT-qPCR) was performed using the StepOne system (Applied Biosystems). The relative expression level of each gene was analyzed by the 2-△△Ct method.

***Statistical analysis***

The normality of cytokine level was evaluated by Kolmogorov–Smirnov test. ANOVA and Kruskal Wallis tests were also used to analyze the differences between the study groups regarding IL 10 and INF-γ, respectively. Kolmogorov-Smirnov, Kruskal Wallis, and Mann Whitney were applied for the gene expression analysis. A log-rank (Mantel-cox) test was applied to compare survival rates. The one-way ANOVA was used to compare the tumor size between the study groups. Kolmogorov-Smirnov and Kruskal Wallis statistical tests were used to compare the groups’ differences in tissue weight. Graph Pad Prism 8 software was used to analyze all the data. The data were presented as mean±standard deviation. *P*-values≤0.05 were considered to be significant. 

## Results

***The confirmation of synthesized nanostructures***

**Figure 2:**
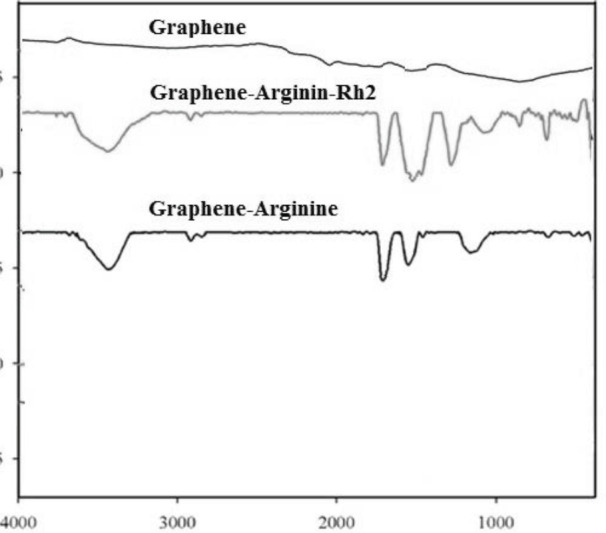
FTIR spectra for synthesized nanostructures, Gr, Gr-Arg, and Gr-Arg-Rh2. Pure graphene with vibrations related to the C-H bond and the hydroxyl group, Gr-Arg, and Gr-Arg-Rh2 with the vibration of the C-N bond and NH-type amine related to arginine, and Gr-Arg-Rh2 with esterification of the carboxyl group of arginine with the hydroxyl groups of ginsenoside Rh2 due to strong peak at 1700 cm^-1^

FTIR: Fourier-transform infrared; GR: Graphene; Gr-Arg-Rh2: Graphene-Arginine-Ginsenoside Rh2; Gr-Arg: Graphene- Arginine

**Figure 3:**
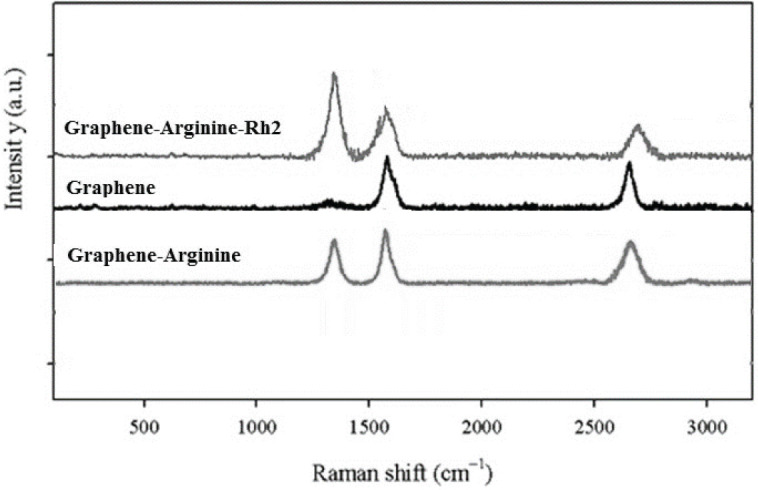
Raman spectra for synthesized nanostructures, Gr, Gr-Arg, and Gr-Arg-Rh2. The high ID/IG ratio confirms the deformation of Gr-Arg and Gr-Arg-Rh2 by functionalization

GR: Graphene; Gr-Arg-Rh2: Graphene-Arginine-Ginsenoside Rh2; Gr-Arg: Graphene- Arginine

**Figure 4:**
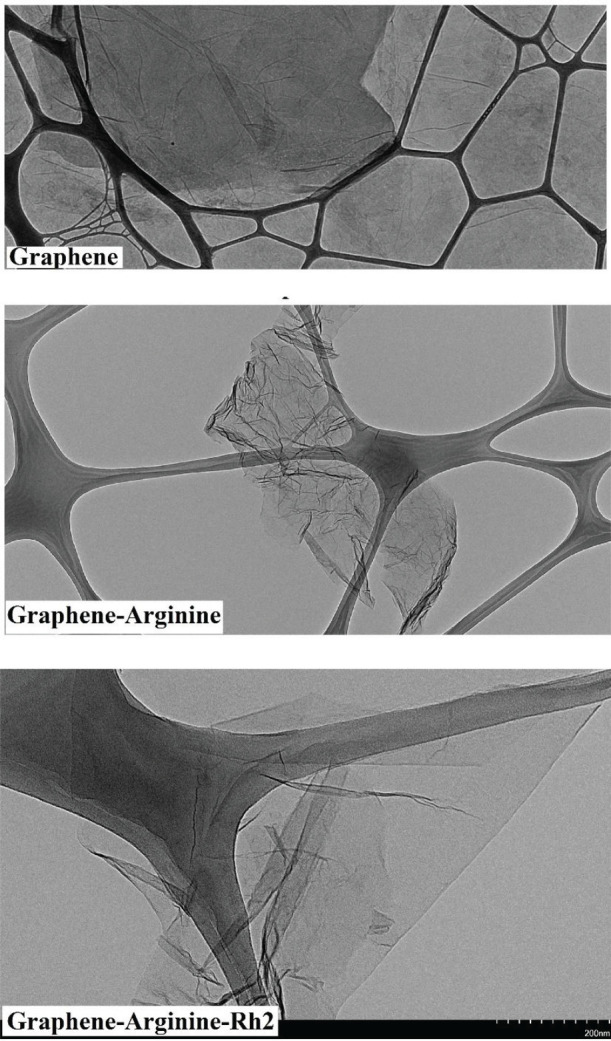
TEM images of Gr, Gr-Arg, and Gr-Arg-Rh2. Wrinkled morphology and folded edges for Gr-Arg and Gr-Arg-Rh2 due to chemical functionalization compared with graphene

GR: Graphene; Gr-Arg-Rh2: Graphene-Arginine-Ginsenoside Rh2; Gr-Arg: Graphene- Arginine

All applied characterization techniques (FTIR, Raman, and TEM) showed the accuracy of the synthesis. The FTIR results suggested that the pure graphene lacked a functional group and came along only with vibrations related to the C-H bond and the hydroxyl group attached to pure graphene. The vibration of the C-N bond and NH-type amine were related to the presence of arginine in the structure of G-Arg and G-Arg-Rh2. The esterification of the carboxyl group of arginine with the hydroxyl groups of ginsenoside Rh2 in the structure of G-Arg-Rh2 was proved by the strong peak at 1700 cm-1 (Figure 2). In Raman spectra, the pure graphene had no sharp D band, but G-Arg and G-Arg-Rh2 had a sharp D band (Figure 3). The high ID/IG ratio in G-Arg and G-Arg-Rh2 confirmed the structural deformation induced by functionalization. TEM results proved the acquired data from FTIR and Raman (Figure 4). 

***Drug loading and release***

**Figure 5:**
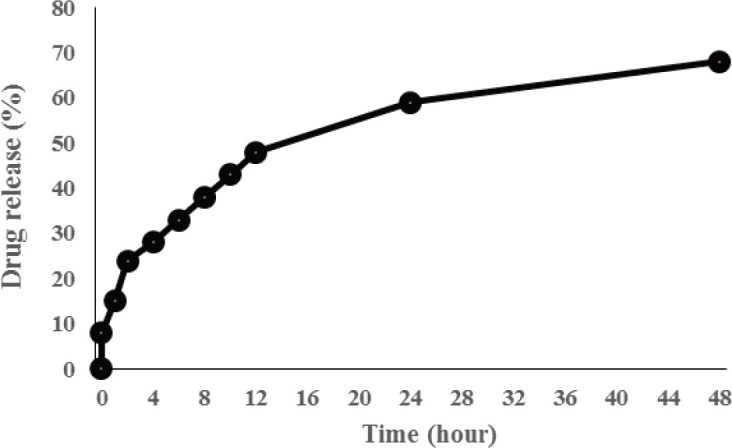
*In vitro* release profile of Rh2 from Gr-Arg-Rh2 in a 48 hr duration. Sixty-eight percent of the drug was slowly released from the designed nanostructures in 48 hr

Drug loading evaluation showed that the encapsulation efficiency of the formulation of G-Arg-Rh2 was 82.4%±5.6. Drug release evaluation also showed that 68% of the drug was slowly released from the designed nanostructures in a period of 48 hr (Figure 5). 

***Biological assessment of the designed nanostructures in the animal model***

*Tumor size and mice survival*

**Figure 6:**
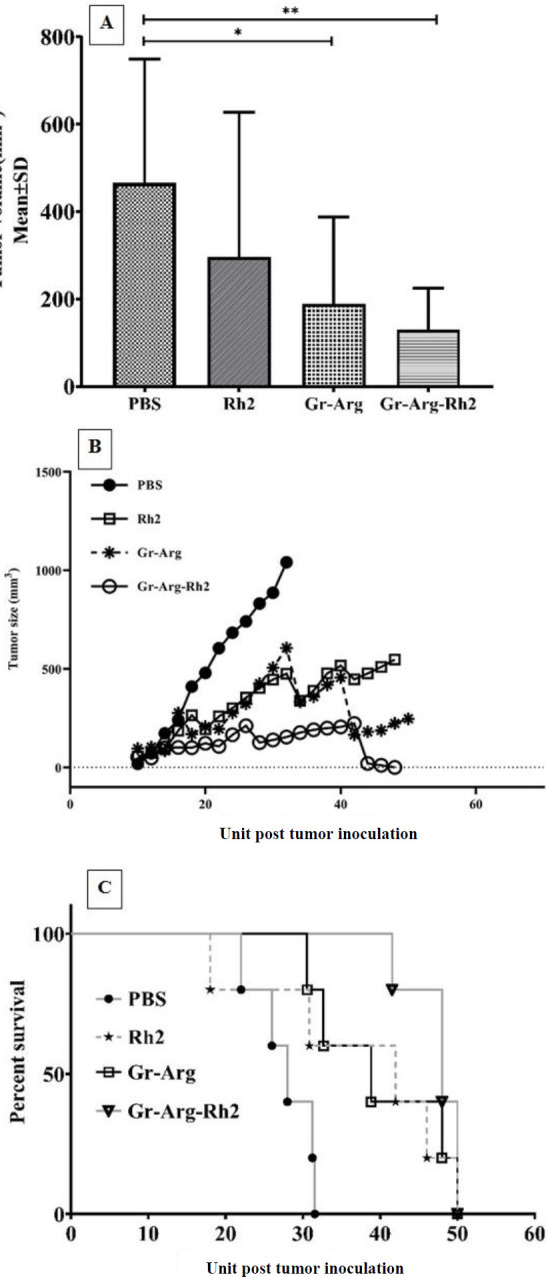
A comparison of tumor volumes (A), tumor growth rate within 50 days (B), and survival percentage (C) in different groups. The gr-Arg-Rh2 group had the lowest volume and tumor growth rate but the highest survival rate

Gr-Arg-Rh2 in the mice significantly inhibited tumor growth compared with Rh2 and control groups (Figure 6). The log-rank (Mantel-cox) survival analysis showed that the survival time was significantly prolonged in those mice that received Gr-Arg-Rh2 in comparison with other groups (*P*<0.0043) (Figure 6). The different formulations and their TTEs, %TGD, %ILS, and MSTs are shown in Table 2. Gr-Arg-Rh2 inhibited tumor growth (TGD = 71%) and the increased duration of the mice survival were more effectively than PBS as a control. The mice in the Gr-Arg-Rh2 group had longer life spans than the control group. A reduction in 100% of the survival rates began on days 22 and 42 in the control and Gr-Arg-Rh2 groups, respectively. The average rates of TTE, MST, TGD, and ILS were higher in the Gr-Arg-Rh2 group than in other groups (Table 2). MST was 28 and 48 days for the control and Gr-Arg-Rh2 groups, respectively. Comparison of the weight of the tumor, liver, lung, and spleen among study groups was shown in Table 3. Gr-Arg-Rh2, Gr-Arg, and Rh2 led to decrease in tumor weight, especially in the Gr-Arg-Rh2 group (*P*-values are 0.006, 0.007, and 0.014 for Gr-Arg-Rh2, Gr-Arg, and Rh2, respectively). The differences in the weight of the liver, lung, and spleen were not significant among the groups (*P*-values are 0.14, 0.061, and 0.15 for the liver, lung, and spleen, respectively). In the control and Rh2 groups, an increase in liver weight was observed.

*Gene expression results*

The *TGFβ* expression levels of spleen tissue were significantly down-regulated in the immunized mice for all drug treatment groups compared with the control group (*P*=0.04). Additionally, a slight increase in *IFN-γ* mRNA was observed in the Rh2 group compared with other groups, but it was not significant. No significant change in terms of *IL10* and *FOXP3* gene expression profiles (*P*>0.05) was observed in the groups. However, the higher and more adverse *IL10* and *FOXP3* mRNA expression levels belonged to the Gr-Arg group in comparison with other groups (Table 4). 

*Evaluation of necrosis of the tumor surface during the therapy*

**Figure 7:**
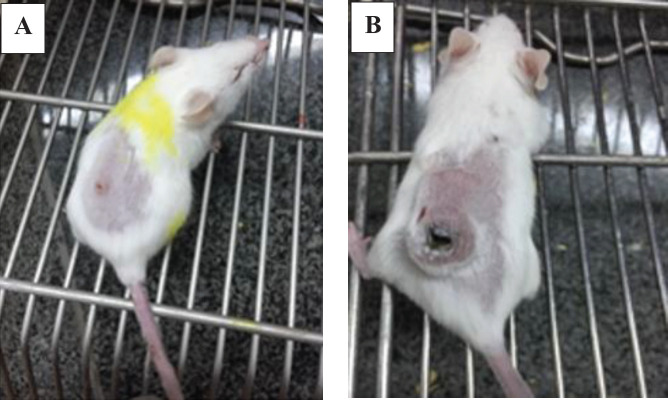
Morphological characteristics of drug therapy in those mice with breast tumors. A) The tumorized mice treated with Gr-Arg-Rh2. In some mice, a large part of the tumor disappeared by necrosis, while in others, the tumor was flattened entirely after ten injections, and only slight necrosis was observed in this group. B) The tumorized mice treated with Gr-Arg. Necrosis was also observed on the surface of the tumor and its body

The first necrosis was observed one week after the first drug injection in the Gr-Arg-Rh2 group. About 87.5% of the mice in this group developed relatively severe necrosis. In one mouse, the necrosis was very intense and a large part of the tumor was destroyed by necrosis after 7 injections. In this group, after 10 injections were performed and 3 mice died, intense necrosis was observed in the whole tumor tissue of two of the mice. In addition, the tumor was completely flat in one mouse after 10 injections and there was slight necrosis on the tumor surface. No tumor was observed after the mice in the Gr-Arg-Rh2 group died. In the Gr-Arg group, 50% of the mice developed necrosis. In one mouse, the necrosis was intense and new necrosis was observed on the tumor surface. Twenty-five days after the first drug injection into these mice, slight necrosis was observed on the body surface. In the Rh2 group, 50% of the mice developed necrosis. In this group, necrosis was not intense. Additionally, after seven injections, one mouse from this group had necrosis on the inner surface of the tumor. In the Gr-Arg-Rh2 group, the tumor size was not very large, necrosis was developed in this group faster compared with the control group. The intensity of necrosis in the Gr-Arg-Rh2 group compared with the other two drug groups indicated a higher number of cancer cells killed in this group (Figure 7).

*Histological evaluation*

**Figure 8:**
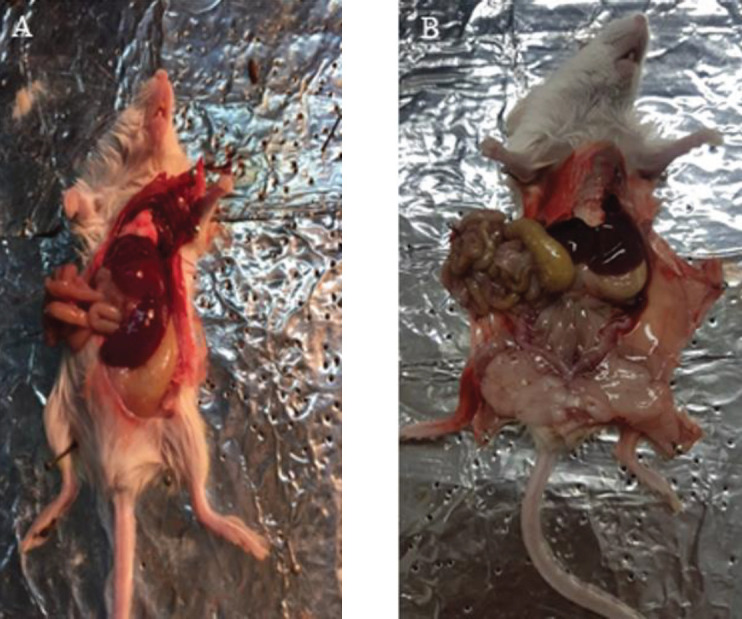
An evaluation of the mice's abdominal viscera. A) The tumorized mice treated with Rh2. In these mice, the volume of the liver, stomach, and intestines increases. B) The normal mice. This figure shows a normal mice's liver, stomach, and intestines volume

According to histopathological results, the liver, stomach, and intestine got very large in two of the mice of the Rh2 group after 7 drug injections (Figure 8). This condition was also seen in the controlled mice. No specificity was observed on the abdominal viscera examinations in the Gr-Arg-Rh2 and Gr-Arg groups. 

**Histopathological assessment of tumor tissue**

**Figure 9:**
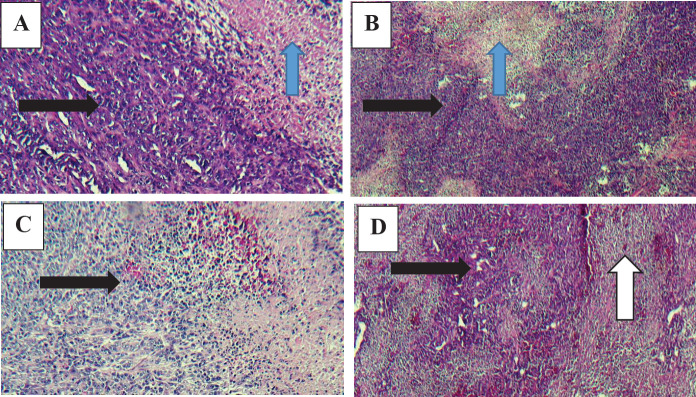
Histopathological slides of tumors (with 40X objective lens). A) Gr-Arg-Rh2 group. The decreased percentage of tumor cells and increased tumor cell necrosis. B) Gr-Arg group. A significant increase of necrosis in tumor cells. C) Rh2 group. A low percentage of necrosis in tumor cells. D) Control group. A very low percentage of necrosis in tumor cells. Blue arrow: necrosis; Black arrow: metastasis; White arrow: Inflammatory cell infiltration

Gr-Arg-Rh2: Graphene-Arginine-Ginsenoside Rh2; Gr-Arg: Graphene- Arginine

A very low percentage of necrosis was observed in the control group compared with the other three groups. In the Rh2 group, no significant issue was observed in tumor histology. The necrosis of the tumors in the Rh2 group was not significantly different from that of the control group. A significant increase in tumor necrosis was evident in the tumors’ histological specimens related to GR-Arg treatment. In the Gr-Arg-Rh2 group, a drop in the percentage of tumor cells and an increase in tumor cell necrosis were observed. The percentage of necrosis observed in this group was higher than that of the Gr-Arg group (Figure 9). 

**Histopathological assessment of lung tissue**

In the control group, 10% involvement of lung tissue cells with the tumor was observed due to metastasis. In this group, strongly acute infiltration of neutrophils was also detected in a part of the tissue. 

In the Rh2 group, acute cell infiltration was well observed in the interstitial space of the lungs and around the arteries. Additionally, mild scattered bleeding was observed. In this group, the metastatic tumor was observed in 7% of the lung cells, which was less than that of the control group. 

In the Gr-Arg group, a tumor was seen in 5% of the sample, which was accompanied by mild alveolar emphysema. This mild destruction observed in the lung was due to tumor metastasis. The percentage of metastasis in this group was much lower than in the control and Rh2 groups. 

**Figure 10:**
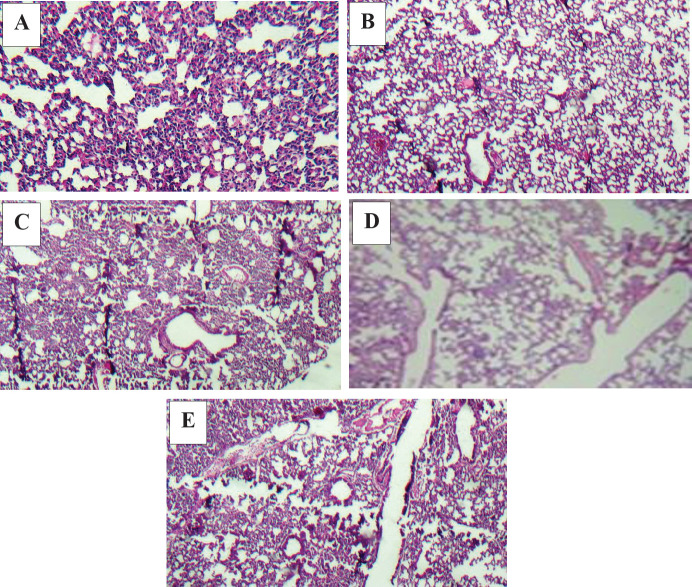
Comparison of histopathological data of lung tumors among study groups. Slides were obtaind using 40X objective lens. A) Gr-Arg-Rh2 group. No metastasis was observed. B) Gr-Arg group. Much less metastasis was observed. C) Rh2 group. Less metastasis was observed. D) Control group. Metastasis was observed. E) Normal mouse. Blue arrow: Infiltration of inflammatory cells in the port space; White arrow: Emphysema in the alveolar spaceGr-Arg-Rh2: Graphene-Arginine-Ginsenoside Rh2; Gr-Arg: Graphene- Arginine

In Gr-Arg-Rh2, lung tissues showed moderate emphysema in the respiratory tract and neutrophil, lymphocyte, and plasma cell infiltration in the interstitial and perivascular space of the lung. No tumor tissue was seen in this group. A noteworthy feature in this group was the absence of a metastatic tumor in any of the lung samples, suggesting the positive effect of Gr-Arg-Rh2 on necrosis augmentation in the primary tumors and preventing metastasis (Figure 10). 

**Histopathological assessment of liver tissue**

Liver tissue showed partial destruction of parenchymal cells in the control group. No metastasis to liver tissue was observed in this group. 

In the Rh2 group, cases of infiltration in neutrophil, lymphocyte, and plasma cells were observed in the portal spaces as multifocal, and also parenchymal cell destruction as feathery degeneration. No liver metastasis was observed in this group. 

In the Gr-Arg group, moderate infiltration of neutrophils was observed in the pore spaces and around the central vein. Mild feathery degeneration of parenchymal cells was also found.

**Figure 11:**
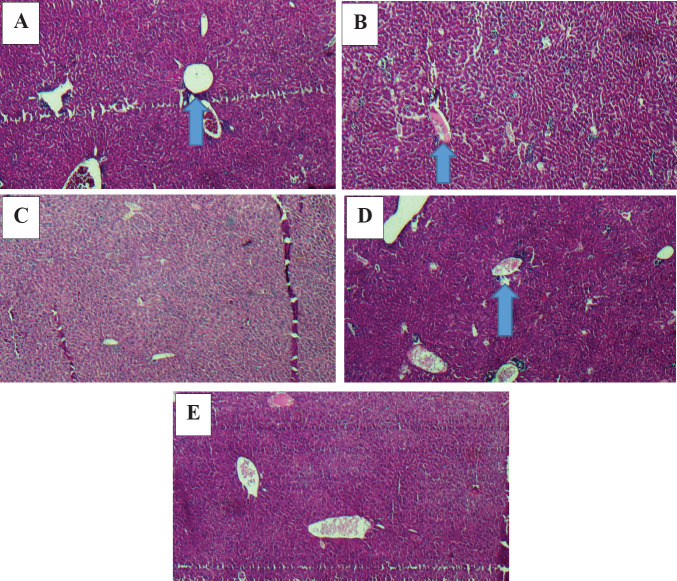
Comparison of histopathological data of liver tumorsamong study groups. Slides were obtained using 40X objective lens. A) Gr-Arg-Rh2 group. A partial degeneration of hepatocytes and acute cell infiltration in the portal spaces and around the central vein. B) Gr-Arg group. A moderate infiltration of acute cells into the spaces of the port and around the central vein. C) Rh2 group. Mild infiltration of acute and chronic cells in portal spaces. D) Control group. Partial destruction of parenchymal cells. E) Normal mouse. Black arrow: An infiltration of inflammatory cells around the central vein; white arrow: An infiltration of inflammatory cells in the port spaceGr-Arg-Rh2: Graphene-Arginine-Ginsenoside Rh2; Gr-Arg: Graphene- Arginine

In the Gr-Arg-Rh2 group, liver tissues demonstrated partial degeneration of hepatocytes and neutrophil cell infiltration in the portal spaces and around the central vein (Figure 11).

**Table 1:** Oligonucleotide primers for gene expression evaluation of FOXP3, TGF-B, IL-10, IFNY, and GAPDH

**Gene**	**Primer sequence (5'-3')**	**Sequence amplified**	**Product size**
***FOXP3***	Forward- CCTGCCTTGGTACATTCGTG Reverse -TGTTGTGGGTGAGTGCTTTG	NM_001199348.1	97 bp
***TGF-β***	Forward -ATTGCTTCAGCTCCACAGAG Reverse –TGTACTGTGTGTCCAGGCTC	NM_011577.2	159 bp
***IL10***	Forward – GAAGGCAGTCCGCAGCTCTA Reverse – GCCTGGCTCAGCACTGCTAT	NM_010548.2	142 bp
***IFNγ***	Forward – AAGTGGCATAGATGTGGAA Reverse – GCTTATGTTGTTGCTGATG	NM_008337.4	160 bp
***GAPDH***	Forward – CACTGCCACCCAGAAGACTG Reverse – CCAGTGAGCTTCCCGTTCAG	NM_001289726.1	147 bp

Foxp3: Forkhead box P3; TGF-β: Transforming growth factor-β; IL10: Interleukin 10; IFNγ: Interferon gamma; GAPDH: Glyceraldehyde-3-phosphate dehydrogenase

Gr-Arg-Rh2: Graphene-Arginine-Ginsenoside Rh2

**Table 2:** Comparison of average of TTE, median survivsl, %TGD, and %ILS among study groups: PBS, Rh2, Gr-Arg, and Gr-Arg-Rh2

Result	Ave of TTE	SD of TTE	Median survival	%TGD	%ILS
PBS	27.75365276	3.539733048	28	0	0
Rh2	37.38345154	11.60988038	42	34.69741032	50
Gr-Arg	40.00991728	7.855655967	38.84033613	44.16090605	38.71549
Gr-Arg-Rh2	47.51029412	3.110770719	48	71.18573374	71.42857

TTE: Time to reach the end-point; TGD: Tumor growth delay; ILS: Increased life span

**Table 3:** Comparison of the weight of tumor, liver, lung, and spleen among study groups: PBS, Rh2, Gr-Arg, and Gr-Arg-Rh2

**Variables **	**Gr-Arg-Rh2** ^a^ (N=8)	**Gr-Arg** ^ b^ (N=8)	**Rh2** ^c^ (N=8)	**PBS** ^ d^ (N=8)	**P-value**
**Tumor weight** **(gr)**	0.34±0.16	0.63±0.18	0.36± 0.21	1.86±0.19	**Pt =0.009** **P** ^a-d^ **=0.006 ** **P** ^b-d ^ **=0.007** **P** ^c-d ^ **=0.014**
**Liver weight** **(gr)**	1.02±0.21	1.11±0.02	1.55±0.26	1.55±0.17	Pt = 0.14 P^a-d^=0.083 **P**^b-d ^**=0.014** P^c-d ^=0.773
**Lung weight** **(gr)**	0.22±0.04	0.19 ±0.008	0.72 ±0.54	0.27 ± 0.05	Pt = 0.061
**Spleen** **Weight** **(gr)**	0.78±0.02	0.41 ± 0.06	0.41± 0.11	0.16 ±0.04	Pt = 0.15

The data was presented as Mean±SEM according to the kolomogorov – Smirnov, and kruskal wallis tests. *P*≤0.05 was considered as significant value

Gr-Arg-Rh2: Graphene-Arginine-Ginsenoside Rh2; Gr-Arg: Graphene- Arginine; PBS: Phosphate-buffered saline

**Table 4:** Comparing the mRNA expression levels of spleen tissue (IL10, INF-Y, TGFB, and FOXP3) among study groups: PBS, Rh2, Gr-Arg, and Gr-Arg-Rh2

**Gene**	**Gr-Arg-Rh2** ^a^ (N=8)	**Gr-Arg** ^ b^ (N=8)	**Rh2** ^c^ (N=8)	**PBS** ^d^ (N=8)	**P-value**
***IL10***	0.50±0.18	5.36±3.03	0.69±0.37	1.11±0.16	Pt=0.43 P^a-d^ =0.38 P^b-d ^ =0.66 P^c-d ^ =0.62
***I*** ***NF-γ***	0.61±0.35	0.68±0.33	1.14±1.1	0.82±0.08	Pt=0.63 P^a-d^ =0.38 P^b-d ^ =0.38 P^c-d ^ =0.4
***TGFβ***	2.31±1.78	1.93±1.20	0.03±0.03	6.98±6.64	**Pt=0.04** P^a-d^ =0.18 P^b-d ^ =1 P^c-d ^ =0.06
***FOXP3***	0.35±0.17	5.42±4.64	0.63±0.32	0.9±0.01	Pt=0.284 P^a-d^ =0.117 P^b-d ^ =1 P^c-d ^ =0.85

Foxp3: Forkhead box P3; TGF-β: Transforming growth factor-β; IL10: Interleukin 10; IFNγ: Interferon gamma; GAPDH: Glyceraldehyde-3-phosphate dehydrogenase; Pt: total. The data was presented as Mean±SEM according to the kolomogorov-smirnov, kruskal wallis and Mann whiteney tests. *P*≤ 0.05 was considered as significant value

Gr-Arg-Rh2: Graphene-Arginine-Ginsenoside Rh2

## Discussion

In this study, Gr-Arg and Gr-Arg-Rh2 were synthesized and their anticancer effect was evaluated in mice with cancer. One of the positive aspects of designed nanostructures, especially Gr-Arg-Rh2, was increasing their dispersion in water. This situation facilitated the evaluation of their biological effect. This drug was easily injected and used intravenously in mice. Covalent functionalization with Arg and Rh2 improved the dispersion of graphene. Functionalization with Arg and Rh2 was carried out by producing semi-stable diazonium ions for the initiation of the radical reaction. Reduced time, strong interaction, and localized heating are the most important properties of the microwave technique in graphene reduction, exfoliation, modifications, and functionalization (24). 

Acquired data showed a sustained and slow drug release from the Gr-Arg bed. Our data are consistent with other similar studies with a difference in the type of loaded drug as well as the interaction type (25-27). 

Gr-Arg-Rh2 inhibited tumor growth and the increased duration of the mice’s survival more than the control. The survival rates in the Gr-Arg-Rh2 group were longer than in the control group. These sets of data suggested that the survival rate in the Gr-Arg-Rh2 group was doubled compared with the control group. Given that, the major cause of death due to cancer is metastasis and the major effect of cancer treatments, such as chemotherapy, is to reduce the metastasis (28), and the survival rate enhancement in Gr-Arg-Rh2 can be related to the reduction of metastasis. Histopathological assessment of lung tissue showed that there was metastasis in the lungs in the control group. Partial destruction of parenchymal cells was also shown in liver tissue in the control group. In Rh2 and Gr-Arg groups, the metastatic tumor was also observed in the lung cells with a lower percentage than in the control group. No metastatic tumor was seen in the Gr-Arg-Rh2 group in both liver and lung tissues. Lung or bone metastasis occurs in more than 60% of patients with breast cancer (29). Therefore, pulmonary involvement in patients with breast cancer has a high prevalence. Drugs that can reduce the rate of this metastasis have a significant impact on the recovery process of breast cancer patients (30, 31). In our study, Gr-Arg-Rh2 showed this significant impact. At the same time, the metastasis process was better controlled in Gr-Arg-Rh2 in comparison with Rh2 alone and Gr-Arg. On the other hand, we found that the side effects of Gr-Arg and Gr-Arg-Rh2 were somewhat greater than Rh2. Based on the previous articles, arginine can enhance tumor sensitivity to anticancer agents (32). This amino acid is also capable of decreasing tumor incidence and overall burden in the early stages of the disease. However, according to various studies, this amino acid is considered a tumor-promoting and tumor-inhibiting agent at the same time (33-35). On the other hand, graphene nanostructures can sensitize the cancer cells to chemotherapy agents by inducing early autophagy events, promoting nuclear trafficking, and developing necrosis (36-38). Necrosis induction in cancerous cells by carbon nanostructures was proven (38). Accordingly, the exacerbation of necrosis can be justified in the Gr-Arg and Gr-Arg-Rh2 groups, especially in the Gr-Arg-Rh2 group. 

Necrosis on the body’s surface may be a sign of drug toxicity. Approximately 10 days after the first drug injection, the control mice developed necrosis. Due to the high metastatic property of the 4T1 cell line, this type of tumor grows rapidly (39). Inhibition of tumor development leads to necrosis. In the Gr-Arg-Rh2 group, the destruction of a large part of the tumor indicated the inhibitory effects of Gr-Arg-Rh2 on the decreasing trend of the tumor size and weight by inducing necrosis. The data indicated that only tumor weight led to a significant difference between the study groups. In general, the differences in the weight of the liver, lung, and spleen were not significant. In the control and Rh2 groups, an increase in liver weight was observed. This weight increase can occur as a result of vein occlusion by cancer cells or metastasis (40). In the Rh2 groups, the mean lung weight was very high. Malignant ascites or accumulation of fluids in the space around the organs in the abdomen is most common in patients with breast, colon, ovarian, pancreatic, uterine, stomach, and intestinal cancer (41). This status leads to weight gain, dyspnea, abdominal swelling, sense of fullness or bloating, sense of heaviness, indigestion, nausea or vomiting, changes in the belly button, hemorrhoids, ankle swelling, fatigue, and loss of appetite (42). In our study, these ascites were observed in the control and RH2 groups. Among all groups, the Gr-Arg-Rh2 group experienced more severe necrosis in tumor tissues. In the ginsenoside group, metastasis occurred in lung tissue, leading to histological damage. This damage was also observed in Gr-Arg-Rh2 and Gr-Arg groups. However, no tumor tissue was observed in the lung tissue of the Gr-Arg-Rh2 group, indicating the efficacy of the drug in the prevention of the metastatic process. In the Gr-Arg group, tumor involvement was observed in a sample of lung tissue. According to the presented results, Gr-Arg-Rh2 led to a more intense induction of necrosis in the tumor tissue. 

All survived mice were discarded after 50 days. Cytokines, such as interferons (IFNs- α, -β, and –γ), interleukins (ILs-2, -6, and -10), and tumor necrosis factor (TNF-α), play an important role in various types of cancer, especially in breast cancer (43). Studies showed that *TGF-β* as a cytokine had a key role in breast cancer progression (43, 44). Therefore, the inhibition of *TGF-β* can block the tumor (45). TGFβ can lead to vessel invasion, promote cancer-associated fibroblasts, develop tumors, increase metastasis, and decrease in survival rate in cancerous patients (46). Thus, reduction of TGFβ expression is considered a key objective in many targeted cancer treatments. The molecular mechanism of TGF-β in inhibiting or progressing the tumor is involved through the intervention and mediation of Smad. So, this pathway has a dual role in tumor progression. Studies showed that the TGF-β/Smad signaling pathway has the main role in tumor promotion in the last phase of cancer. Therefore, in this phase of the disease, any drug that has an effective anti-cancer effect leads to a decrease in TGF-β gene expression and stops the TGF-β/Smad signaling pathway (47, 48). In our study, there was a significant reduction of *TGFβ* expression level in the Gr-Arg-Rh2 group, compared with the control group. This change in *TGFβ* expression shows the beneficial effect of the designed drug in preventing tumor progression and metastasis.

 IL-10 as an anti-inflammatory cytokine prevents the pro-inflammatory functions of antigen-presenting cells (49). This cytokine is especially involved in breast cancer (50). IL-10, as a multifunctional cytokine, leads to promotion of tumor proliferation and metastasis. However, this cytokine is considered both a tumor-promoting and -preventing factor. In more than 50% of breast cancers, higher IL-10 expression was reported. IL-10 can stimulate tumor proliferation and metastasis via suppression of the proliferation and activity of T cells (51). Thus, the serum levels of IL-10 is high in breast cancer. On the other hand, breast cancer drug resistance can occur after enchantment of IL-10 secretion by tumor-associated macrophages. Jiang et al. showed two molecular pathways, IL-10/IL-10R and IL-12/IL-12R can change the tumor response to chemotherapy drugs (52). A decrease in IL-10 expression was seen in Gr-Arg-Rh2 and Rh2 groups compared with the control group. An increase in IL-10 expression was seen in the Gr-Arg group compared with the control group. However, these changes were not statistically significant. This result shows that the changes in IL-10 gene expression are caused by Rh2 in Gr-Arg-Rh2.

*Forkhead box P3* (*Foxp3*) also has a similar effect on cancer cells (53). The enhancement of *Foxp3* expression leads to tumor progression by activating the Wnt/β-catenin signaling pathway (54). *Foxp3* levels in the peripheral blood and tumor specimens of cancer patients are associated with tumor progression and poor prognosis (55). A decrease in *Foxp3 *expression was seen in Gr-Arg-Rh2 and Rh2 groups compared with the control group. An increase in *Foxp3*expression was seen in the Gr-Arg group compared with the control group. However, these changes were not statistically significant. This result shows that the changes in *Foxp3 *gene expression are also caused by Rh2 in Gr-Arg-Rh2.

In contrast, *IFNγ* can prevent tumor progression by inhibiting the tumor cell cycle and induction of apoptosis and necroptosis (56, 57). In breast cancer, pleiotropic cytokines enhance the expression of cell cycle inhibitor proteins, including p27Kip, p16, and p21. Accordingly, any drug combinations that could reduce the levels of *TGF-β, IL-10*, and *Foxp3* and increase the level of IFNγ, would have a potent inhibitory effect on tumor growth (44, 58-61). The best condition occurred in the Rh2 group. Enchantment of IFNγ expression in Rh2 groups was higher than in other groups. In general, the changes in the expression of the examined genes in the Gr-Arg-Rh2 group were more suitable than the other groups, and these changes were in line with the positive effect of Gr-Arg-Rh2 in inhibiting tumor growth in the treated mice.

## Conclusion

According to the results, Gr-Arg-Rh2 decreased tumor size, weight, and growth and increased the survival time of the mice with cancer. A significant decrease in TGF-β gene expression was also observed in the Gr-Arg-Rh2 group. Generally, Gr-Arg-Rh2 led to suitable changes in the expression of the examined genes along with tumor suppression. According to histopathological results, Gr-Arg-Rh2 also caused necrosis in tumor cells and well-inhibited metastasis. In addition, the lowest level of side effects was seen in this group. So, this study suggested Gr-Arg-Rh2 as a tumor inhibitor. 

## Authors’ Contributions

All authors contributed to the manuscript’s design, writing, and review. SDF Contributed to experimental sections, including material synthesis and animal treatment. FF Conducted the synthesis of nanomaterials and biological tests. MDM Carried out drug treatment and gene expression sections. FZ Contributed to MTT assay, hematological assessment, and animal treatment. SM M handled animal treatment and statistical analysis of data. MV did a histopathological study of tumors and normal tissues. HS did a histopathological examination, Pharmacokinetics, and article editing. HZZ Led the laboratory operations and fieldwork for the entire project and managed and supervised the experiments and results. All authors were involved in checking the manuscript text.

## Conflicts of Interest

The authors declare no competing interests.
